# Using Textural Analysis of Thermal Imaging to Predict Healing Status of Diabetes-Related Neuropathic Foot Ulcers: Protocol for a Co-Design and Longitudinal Study

**DOI:** 10.2196/69793

**Published:** 2026-02-26

**Authors:** Kate Waller, Rajna Ogrin, Quoc Cuong Ngo, Barbara Polus, Samantha Hanna, Aye Nyein Tint, Richard J MacIsaac, Anna Galligan, Elif Ilhan Ekinci, Dinesh Kumar

**Affiliations:** 1 Department of Podiatry and High Risk Foot Service, St Vincent’s Hospital Melbourne, Melbourne, Victoria, Australia; 2 Support Services Office, Bolton Clarke, Forest Hill, Victoria, Australia; 3 Australian Centre for Accelerating Diabetes Innovations (ACADI), Melbourne Medical School, University of Melbourne, Melbourne, Victoria, Australia; 4 School of Engineering, RMIT University, 124 La Trobe St, Melbourne, Victoria, 3000, Australia, 61 (03) 9925 2000; 5 Department of Podiatry and High Risk Foot Service, Austin Hospital, Heidelberg, Victoria, Australia; 6 Department of Endocrinology, Austin Health, Heidelberg, Victoria, Australia; 7 Department of Endocrinology and Diabetes, St Vincent’s Hospital Melbourne, Melbourne, Victoria, Australia; 8 Department of Medicine, Melbourne Medical School, University of Melbourne, Melbourne, Victoria, Australia

**Keywords:** diabetic foot, foot ulcer, wound healing, thermal imaging, textural analysis, lower extremity

## Abstract

**Background:**

Diabetes-related foot ulcers (DFUs) are common in people with diabetes and a major cause of poor quality of life and disability. If not treated in a timely and appropriate way, DFUs may result in prolonged hospitalization and amputation. Currently, methods to predict the healing trajectory of DFUs lack accuracy. Thermal imaging has been proposed to overcome these limitations but has been unable to accurately predict delayed healing of DFUs in the early stages of ulcer management. This project aims to ascertain whether textural analysis of a thermal image can predict the healing trajectory of DFUs.

**Objective:**

The study aims (1) to co-design an accurate, fast, easy-to-use, computer-aided, nontouch test to predict DFU healing trajectory using texture analysis of thermal imaging that is fit for purpose and acceptable to both, those being tested and users of the device and (2) to validate whether textural analysis of thermal images can accurately predict healing of DFUs at week 12 from an image taken at week 1.

**Methods:**

This project will be undertaken in 2 phases: Phase 1, co-design and development of the software prototype; and Phase 2, technology validation. Phase 1 requires a participatory action, co-design approach, engaging clinicians and biomedical engineers in a facilitated focus group and email communication. Interviews with adults living with diabetes and a DFU or who have a history of a DFU will be undertaken to understand their information needs about the device and its findings. Phase 2 will be a longitudinal observational study of 120 adults living with a DFU over a 12-week period. Demographic and other data that have been shown to impact wound healing will be collected at baseline, including participant age, gender, wound size, wound duration, and biomedical markers. Thermal and standard red, green, and blue images will be taken at weeks 1, 2, and 12. Wound textural features will be entered into a Bayesian neural network to identify the healing trajectory.

**Results:**

Phase 1 has been completed with biomedical engineers and clinicians from High Risk Foot Services at 2 hospitals in Melbourne, Australia. Nine clinicians participated in a co-design focus group, and 6 clinicians communicated via email. Four themes were identified: insights for sector use, how to document and use the device, legal implications, and workflow requirements. Interviews with 4 people living with diabetes were undertaken, with data to be analyzed thematically. Phase 2 data collection will be completed by April 2026.

**Conclusions:**

The study aims to co-design, test, and validate an accurate, feasible, and acceptable device to predict the healing of DFUs at week 12 from an image taken at week 1. This has the potential to assist clinicians in making informed and timely decisions for instigating adjuvant therapies, thereby improving healing and preventing lower extremity amputations.

## Introduction

### Background

Diabetes affects an estimated 463 million people worldwide, a number expected to increase to over 700 million by 2045 [1]. An estimated 131 million people (1.8% of the global population) had a diabetes-related lower extremity complication (neuropathy, foot ulcer, and amputation) in 2016 [2], and the prevalence of diabetes-related foot ulcers (DFUs) worldwide is approximately 6.3% [3], with estimated costs between US $3999 to US $26,000, with high variability due to heterogeneity of research methodologies [4].

Each year in Australia, diabetes-related foot disease causes an estimated 28,000 hospital admissions, 4500 lower extremity amputations, and 1700 deaths, and costs the Australian health system AU $1.6 billion (US $1,073,696) annually [5-7]. People with DFUs have high rates of delayed wound healing and increased rates of disability, cardiovascular disease, lower extremity amputation, and mortality [8].

Reviewing real-world data, as many as 55% to 70% of all DFUs exhibit delayed healing [9]. Predictive knowledge regarding whether an ulcer is healing at the expected trajectory is crucial for timely clinical decisions, as early intervention in ulcers that are not healing can prevent lower extremity amputation [10,11]. It has been suggested that implementing adjuvant therapies at an early stage in wounds that exhibit delayed healing may reduce healing times and other complications of wounds, including amputations [12].

Imaging modalities have been proposed for the assessment of wounds and have the advantage of being noninvasive, noncontact, and providing real-time digital assessment [13]. Conventional imaging methods, such as magnetic resonance imaging, computed tomography, and positron emission tomography, are used to detect infection or bone involvement in DFUs [14]. However, these techniques are not well-suited for routine outpatient care or for predicting wound healing trajectories, as they are constrained by factors such as radiation exposure (tomography computed and positron emission tomography), prolonged acquisition times, and limited accessibility [15-18]. Recent advances in noninvasive imaging technologies have broadened the range of approaches available for DFU assessment and monitoring, with growing interest in the use of standard red, green, and blue (RGB) imaging, thermal imaging, and hyperspectral imaging to provide practical, safe, and scalable solutions for evaluating wound status and healing potential.

Standard RGB images are used to analyze the area [19] or the shape of an ulcer [20,21]. While these have the advantage of using cameras that are located in currently available digital devices, such as a smartphone, the color and texture of the skin can affect the outcomes of such a technique [22]. The advancements in artificial intelligence (AI) and deep learning have overcome some of the limitations, but there are large differences between the performance of different imaging modalities in assessing wounds in different studies [23].

Hyperspectral imaging is a modality for the assessment of the spectrum of reflected light and has been proposed for the assessment of DFUs and other wounds [24]. However, the performance is dependent on factors such as lighting conditions and the skin color of the person who has the wound [24]. Further, the cost of hyperspectral imaging may be prohibitive, estimated at AU $50,000 (US $33,553) per unit. Furthermore, the process is slow and is computationally expensive, which can be challenging in a clinical setting [24].

In recent years, thermal imaging has been investigated to predict the healing trajectory in DFUs [13]. Thermal imaging enables the detection of temperature differences, quantifying sensitive changes in skin temperature that are associated with pathological changes such as soft tissue inflammation of the skin, subsequent breakdown, and infection of ulcers [25]. Isothermal maps of thermal images to assess changes in the area of DFUs have been used to successfully predict healing at week 4 [13]. The results, however, were highly sensitive to ambient conditions such as the temperature [13,26]. There is currently no dedicated research focused on identifying delayed healing DFUs solely at the first presentation.

Research into venous leg ulcers has shown that textural features of thermal images can provide a suitable binary classification of wounds to determine healing versus delayed healing wounds [27]. Textural features of thermal images using machine learning and AI-based technology were investigated [27,28]. This approach involves machine learning, training the model from wounds recorded and analyzed with known labels of healing within a 12-week period and was successfully proven in venous leg ulcers, overcoming the environmental limitations [27]. The advantage of this technique is that it is not sensitive to the absolute values of the temperature and thus not affected by ambient conditions or skin color [27]. Further, it has been shown to predict the healing of venous leg ulcers with 1 image, with a sensitivity of 78% [27]. This is relevant for the device to have global usability, regardless of large climatic variations and access to specialist care being limited.

### Study Aims and Objectives

The aims of this research, based on previous co-design workshops, are (1) to co-design and develop an inexpensive, fast, AI-based computer-aided, noninvasive test to predict DFU healing trajectory using a thermal imaging device enabling textural analysis that is fit for purpose and acceptable to both those being tested and users of the device; (2) to validate whether textural analysis of thermal images can accurately predict healing of DFUs at week 12 from an image taken at week 1.

## Methods

### Study Overview

This study will be undertaken in 2 phases: Phase 1 is the co-design and development of the prototype, a software-based medical device; and Phase 2 is technology validation. The study reporting is guided by the STROBE (Strengthening the Reporting of Observational Studies in Epidemiology) checklist for cohort studies [29] (Checklist 1).

### Setting

This research will be undertaken in Melbourne, Australia, at High Risk Foot Services of 2 tertiary hospitals. These high-risk foot services provide multidisciplinary care for patients with diabetes-related foot complications. The prevention and treatment of diabetes-related foot disease depends upon a well-organized team of integrated clinicians from various disciplines who use a holistic approach to the assessment and treatment of a foot ulcer and its contribution to multiorgan disease [30].

### Phase 1: Co-Design and Improvement of Prototype

#### Study Design

Co-design entails the active engagement of all stakeholders, drawing on their unique experiences to shape the design of tools, products, or programs [31,32].

**Figure 1.**
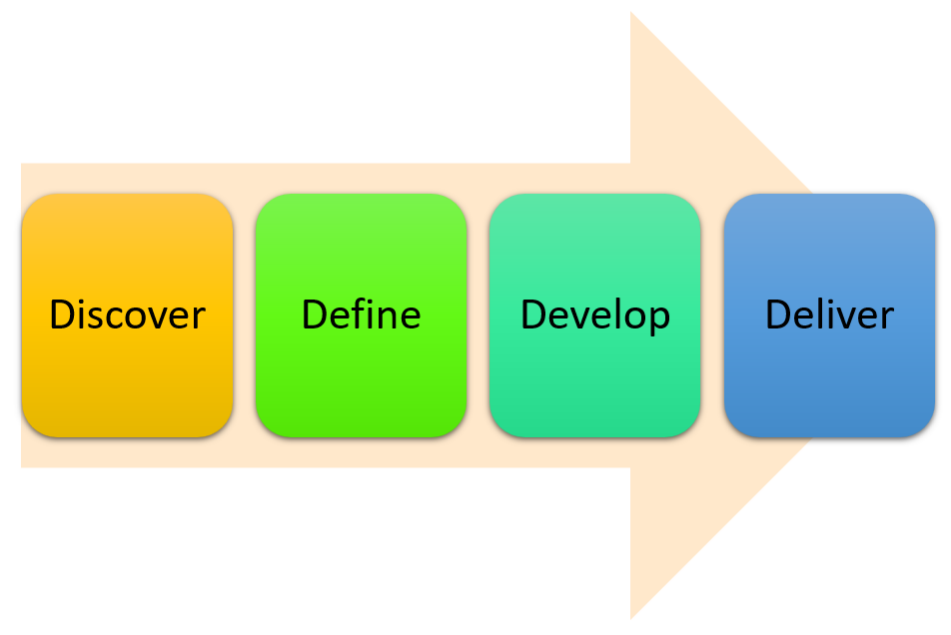
A co-design framework for this study.

Phase 1 used a co-design approach with a combination of the Design Council’s Double Diamond framework [33] and work conducted in New Zealand [31] (Figure 1) to explore participants’ views, knowledge, and perceptions regarding the use of imaging AI technology to collect data to enable the prediction of DFU healing and develop mitigation strategies to address these.

- *Discover:* Undertaking literature searches of (a) the variables that impact the ability of DFUs to heal and (b) devices used to predict the healing of DFUs.
- *Define:* Refining the literature findings to generate a near-final list to include in the device regarding device features and variables to include in the validation of the device.
- *Develop:* Generate a first prototype and then work with key stakeholders to refine the device features and variables to be included as part of the device.
- *Deliver:* Test and validate in the pilot.

#### Participants

Inclusion criteria for key stakeholders are: (i) clinicians working with people who have DFUs, (ii) adults living with diabetes who have a history of or a current foot ulcer, and (iii) biomedical engineer research team members.

#### Recruitment

Clinicians of participating High Risk Foot Services were identified by the research team and invited either in person, via email, or by telephone to participate in the focus group sessions. Existing adult patients of the participating High Risk Foot Services who have diabetes and current or history of a foot ulcer were informed of the research by clinicians working in these services and directed to the advertisement at participating hospitals. All the biomedical engineers from the research team participated in the study. All participants provided informed consent to participate in the study.

#### Data Collection

Facilitator-guided (RO, DK) face-to-face group discussion was conducted with clinicians working with people who have DFUs (n=9) and biomedical engineer research team members (n=2). The session was held in a meeting room at a participating hospital during a time that suited participants, and a working lunch was provided. The device was explained, and a prototype of the software-based medical device was shown to the participants. The meeting was conversational, and participants were asked their opinion on the device concept and what would be needed to use the device at their workplace. Notes were taken of the discussion by 2 scribes (BP and QCN). Clinicians were also asked about their employment position, level and type of education, duration in the field, and duration with the current health service. Follow-up information was obtained through emails between the biomedical engineers and clinicians.

Individual interviews have also been undertaken by a research team member with experience in qualitative research (RO) with 4 adults living with diabetes who had a history of (n=1) or a current foot ulcer (n=3). Interviews were undertaken in a private area within the hospital. The participants were informed about the purpose and design of the device, and participants were asked to share their thoughts on the design of the device and the format and information of the report from the device. They were also asked for their thoughts on the process of collecting the image and the presentation of the information obtained by the device. The interviews were recorded on Microsoft Teams, transcribed using the embedded AI within the program, and validated by a researcher (RO). Participants were also asked about their education level, duration of diabetes, gender, and history of any current or previous lower limb wound.

#### Co-Design Data Analysis

The research team (RO, DK, BP, QCN, and EIE) reviewed the data generated from the co-design to become familiar with the content and key ideas that were raised during the co-design session. These team members met as a group to discuss what concepts they identified from their respective perspectives, coming to a consensus on core themes, as well as additional questions to be raised subsequently for future research. A structured summary was developed by 1 member of the team (RO), with input from the other research team members (DK, BP, and QCN), where the final draft was shared with participants for their input. Further discussion with the research team followed, considering the additional input from participants, and a final summary including next steps was generated, then shared with the participants.

Decisions to incorporate co-design findings in developing the prototype device for use in Phase 2 were made by the biomedical engineers, considering whether the recommendations were (1) a priority to the participants, (2) would make a significant improvement for data collection, and (3) feasible to incorporate, that is not overly costly or require considerable resources, such as engineer time.

#### Interview Data Analysis

The researchers will use an interpretive paradigm, utilizing a relativist approach to ontology and a constructivist approach to epistemology. Reflexive thematic analysis, both deductive and inductive approaches, will be used [34]. Deductive codes are based on usability, accessibility, clinical integration, and support needs. Inductive codes emerge through open coding of the transcripts, which capture concerns and suggestions. To generate key themes and concepts, a researcher will review the interview transcripts to become familiar with the content and consider what key ideas were raised during the interviews. The data will also consider specifics around device use and how it can be best explained by health care providers.

#### Improvement of the Prototype

The current version of the prototype of the thermal imaging device with the smartphone app has been named Thermul. The Thermul will be shown to and used by the stakeholders during the co-design workshops. The feedback will be obtained from these workshops and will be used to modify the device such that it is better accepted by the users and people living with the disease. The major factors that require modification will be identified, and the modified device will be ready for the next discussion with the stakeholders.

### Phase 2: Validation of the Technology

#### Study Design

Participants will be enrolled in this longitudinal observational study for 12 weeks to test whether differences between textural features of thermal images of DFUs can predict ulcer healing at 12 weeks from an image taken at week 1.

#### Participants

Inclusion criteria (Textbox 1) will be adults with diabetes and peripheral neuropathy with a loss of protective sensation and a foot ulcer of any duration with Grade 0 WIfI Wound Infection and Grade 0 WIfI Ischemia classifications [35]. Loss of protective sensation will be determined by the detection of the 10 g (5.07 Semmes-Weinstein) monofilament, according to the sensory foot examination described by Schaper et al [30]. People with signs or symptoms of foot ulcer infection (WIfI infection grade 1‐3), and peripheral arterial disease (WIfI ischemia grades 1‐3) [35] will be excluded. Participants must have sufficient English and cognition (Rowland Universal Dementia Assessment Scale score >22 [36]) to understand the study information and be available to participate over the study period of 12 weeks.

Textbox 1. **Inclusion criteria of the study are as follows:**

- Diabetes diagnosis
- Peripheral neuropathy with loss of protective sensation
- Foot ulcer of any duration
- Wound, Ischemia, foot Infection (WIfI) Wound Infection grade 0 (uninfected)
- WIfI Ischemia grade 0 (Ankle-Brachial Index ≥0.8 or ankle systolic pressure >100 mmHg or toe pressure ≥60 mmHg).

#### Recruitment

Patients attending the participating High Risk Foot Services who fulfill the study inclusion criteria will be invited to participate in the study.

#### Sample Size Calculation

Based on published epidemiological data [37-41] and supported by other pilot work [13], the expected population proportion of true positive ratings for wound healing within 12 weeks is 0.7. Using the goodness-of-fit approach [42], recruiting 120 patients would yield 0.8 power to reject the null hypothesis (substantial agreement, Kappa=0.7) in favor of the alternative hypothesis of almost perfect agreement (Kappa=0.9) using a 2-sided significance threshold of 0.05. For the primary analysis, the concordance between the model and the true status of wound healing at 12 weeks will be evaluated using Cohen Kappa measure for statistical agreement and reported with a respective 95% CI. In addition, sensitivity, specificity, area under the receiver operating characteristic curve, and diagnostic odds ratio will be reported to evaluate the device’s performance as a diagnostic test for determining the true wound-healing status at 12 weeks.

Participants will not be matched but will rather act as their own control. Areas of normal skin from the same image will be compared with areas of ulceration in the digital images. The differences between these 2 sites and changes in these sites over time will enable us to determine if a wound is improving or deteriorating with time.

#### Thermal Imaging Device

The imaging methodology is based on the work of Ngo et al [27], with the base camera specifications shown in Table 1. The device, named Thermul, AU $1400 (US $939.48), takes a thermal and standard RGB image simultaneously in a physically fixed configuration. This ensures that the 2 images do not display measurable parallax. The FLIR One device (thermal camera) is calibrated before each use using the company-provided calibration function. The RGB image with higher resolution is taken from the built-in camera of the DOOGEE (Android) phone. Calibration is undertaken by placing a fixed-size marker next to the wound. Image registration is performed by software that tests the registration of the 2 modalities based on the wound edges. While previous works have used temperature as a feature, our approach uses texture, and this approach has been designed to overcome the ambient temperature limitation present when using thermal imaging of the foot.

Photos are being taken in normal consulting clinic rooms. The equipment has been designed to require minimal training from the user, allowing it to be used much like a regular smartphone. Each staff member is trained to use the device using a minimum of 2 examples, to ensure they are confident to record images independently.

**Table 1.** Base camera specifications prior to co-design.

Camera specifications (minimum)	Thermal camera	Color camera (standard RGB)^a^
Brand options	FLIR or similar	DOOGEE
Model number options	One Pro or similar	S96Pro or similar
Image size (Pixels)	160×120 (thermal resolution) or higher	4000×3000 or higher
Physical dimension (L×W×H) options	68×34×14 mm	167×81.4×15.5
Spectral range	8-14 μm	RGB
Weight	≤40 g	310 g
Power	Battery-USB^b^ data/charging port	Battery-USB data/charging port
Mode of action	Snapshot	Snapshot

a RGB; red, blue, and green.

b USB: Universal Serial Bus.

#### Data Collection

Participant demographic information includes age, gender, socioeconomic factors, and years of formal education. Factors related to the healing of DFUs will also be collected, the specifics of which will be informed on the finalization of the findings of Phase 1, and may include: body mass index, smoking history, retinopathy, types of diabetes treatment, types of DFU treatment, ulcer duration, area of ulcer, depth of ulcer, location of ulcer, infection, blood glucose level, and estimated glomerular filtration rate [43-48]. This information may be revised at a later stage and, if appropriate, will be added to the prediction model.

**Figure 2.**
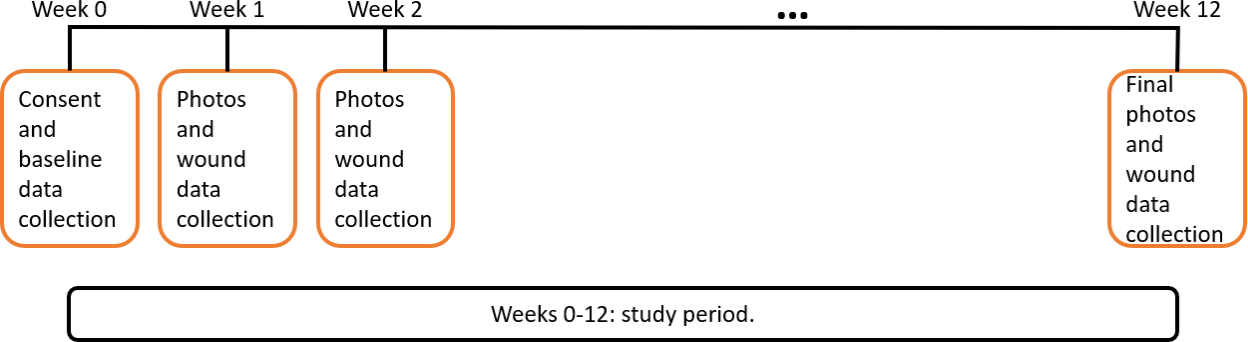
Study timeline.

The following data are collected at baseline, 1-, 2-, and 12-week follow-up visits for all participants (Figure 2):

1. Any signs and symptoms of foot ulcer infection as per WIfI Classification [35].
2. After the ulcer dressing has been removed and prior to any wound hygiene, cleansing, or debridement, the following images will be taken (detailed specifications in Table 1):
  - 160×120 pixel thermal images using the FLIR One Pro.
  - 4000×3000 pixel standard (RGB) using a DOOGEE S96Pro camera.
  - Wound dimensions (length, width, and depth in millimeters).

**Figure 3.**
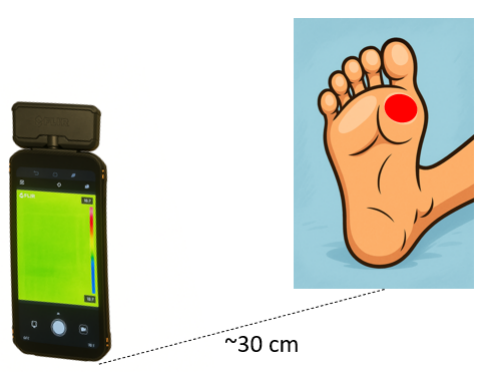
Thermal data collection setup.

Images are taken by trained research and clinical staff, using the following method:

1. The participant is sitting or lying in the bed in a position to ensure that the ulcer is clearly visible.
2. As the 2 cameras are fixed together, the angle of capture of both cameras is the same, with the focus on the ulcer (Figure 3).
  - Both cameras are snapshot cameras, so the images are captured in a single shot, virtually instantaneously.
  - In cases of multiple ulcers in 1 foot or in both feet, each ulcer is considered as an independent ulcer.
  - The imaging uses a fixed-size marker that allows for recording independent images for each ulcer .
3. The images are taken twice while the participant is barefoot.
  - The first image is taken immediately after the wound dressing is removed, and the second approximately 15 minutes later, following all wound hygiene and debridement, as required and part of the usual wound care.

### Statistical Analysis

Thermal images will first be analyzed to obtain textural features using the gray-level co-occurrence matrix. This method helps to extract clinically relevant information from thermal images of wounds by quantifying the spatial distribution of temperature variations rather than relying solely on mean temperature values. Wound tissue often exhibits heterogeneous heat patterns due to differences in vascularization, inflammation, infection, or necrosis, and these subtle variations are best captured through gray-level co-occurrence matrix-derived features. For example, contrast highlights sharp thermal gradients at wound borders, homogeneity reflects the degree of uniform healing, entropy indicates irregularity or randomness associated with pathological processes, and energy captures the smoothness of well-healed tissue [49]. Following this, textural features will be used to identify the healing trajectory of the wound. The textural features will first be reduced using principal component analysis, and the selected features will be classified using different machine learning models, such as logistic regression and Bayesian neural networks, to obtain the probability of healing. The machine learning models will be trained on outcome labels (healed or unhealed) determined by podiatrists at the 12-week clinical evaluation. Two cross-validation approaches will be used: leave-one-out and *k*-fold. The receiver operating characteristic curve will determine the threshold for optimizing the training of the model for the prediction of unhealed wounds. The hypothesis is that factors such as size and infection history should not alter the texture of the thermal image of the wound, and hence should not play a role in the assessment by this modality. The patient demographic information will be analyzed using multivariate logistic regression.

### Ethical Considerations

This study follows the Declaration of Helsinki for human participants, which considers respect for the individual, his or her right to self-determination, and the right to make informed decisions regarding participation in research [50]. Human Research Ethics project approval has been obtained from the Austin Health Human Research Ethics Committee (HREC/92993/Austin-2023) and from St Vincent’s Hospital Melbourne (2024/PID00243). Written consent is obtained from all participants.

Participant privacy and confidentiality were strictly maintained throughout the study. All data were deidentified prior to analysis, stored on secure, password-protected servers at RMIT University, and accessed only by authorized members of the research team. No personally identifiable information is included in any publications.

## Results

Data collection for Phase 1 commenced in February 2024, and co-design workshops with clinicians, biomedical engineers, and clinical researchers concluded in June 2025. Co-design data collection with adults living with diabetes who have a history or a current foot ulcer continues and is expected to conclude in April 2026. Phase 2 commenced in May 2024 and is also expected to conclude in April 2026.

Co-design was undertaken with 9 clinicians (podiatrists, prosthetist-orthotists, and an endocrinologist), 2 biomedical engineers, and 2 clinician researchers, with follow-up information obtained from 6 clinicians.

Participants identified the practical aspects to support device use in 4 key themes: (1) insights for sector use; (2) how to document and use the device; (3) legal implications; and (4) workflow requirements. These are outlined in Textbox 2.

Textbox 2. **Practical aspects to support device use.**

Insights for sector use

- Device more useful for primary care/community health and regional/rural areas where expertise is limited
- Suggest data from the device can supplement existing diagnostics in high-risk foot clinics, not replace them

How to document and use device

- Percentage prediction of healing—refer/not to expert care
- To inform the person regarding the need for referral, if required

Legal implications

- What if the device suggests favorable results and the opposite happens?
- What if the device is used by untrained professionals?

Workflow requirements

- Battery charging duration; more robust device; multiple wounds
- How to ensure the taken image is viable—visual feedback
- Report generated by device—content needs further discussion

## Discussion

### Principal Findings

This study aims to co-design, test, and validate a feasible and acceptable device that can accurately predict the healing of neuropathic DFUs. Clinicians provided guidance on practical aspects to enable optimal device use in the clinical setting. The next phase involves collecting images, and a computer model will analyze those wounds that have healed within a 12-week period. The device will use textural analysis and machine learning to accurately predict the healing of DFUs at week 12 from an image taken at the first visit.

Many health care technologies have been developed, yet very few are used in routine clinical care due to limitations in the device’s usability and technologies not catering to the pressing needs of end users [51]. This study is strengthened by engaging with key stakeholders in the early part of prototype development. We anticipate this early engagement will lead to a device that is fit for purpose and more likely to be used by the target clinicians upon validation and release to market [51].

Co-design approaches are used in health care innovation to harness the ideas and insights of key stakeholders, including consumers, to ensure that what is co-designed is more likely to meet the needs of the end user(s) and be feasible to use in target context(s) [52]. Co-design involves working collaboratively in partnership with key stakeholders, based on an equal and reciprocal relationship, to develop ways of doing things that truly meet people’s needs and preferences [53]. As such, it extends beyond traditional approaches to stakeholder engagement, such as informing, which involves sharing information about an intended service or intervention with no feedback sought; and consultation, which involves seeking feedback on an intended service or intervention with the intention of making changes based on responses [54].

This study will develop a software-based nontouch device to detect the healing status of the wound in the first presentation. The identification of variables that can be used in ulcers that have a greater likelihood of healing will enable the generation of a machine learning algorithm. Once this algorithm is developed, further research can be undertaken on ulcer types that have a limited capacity to heal to refine the algorithm for a broader range of DFUs, including ischemic and neuro-ischemic ulcers.

This technology has overcome a number of limitations identified in previous studies [13,24,26-28], including faster speed of image capture, calculation of image data, and reduced cost compared to other diagnostic devices. Ambient temperature, lighting, and skin color and texture also do not influence thermal image quality. This technology is also suitable for investigating the ulcers of any duration or stage of healing.

### Conclusions

The findings of this study, a combined co-design and longitudinal study, will develop a device aimed at predicting the healing of DFUs. The study will determine if textural characteristics from thermal images of neuropathic DFUs can identify which ulcers will not heal by 12 weeks. If successful, the device may be used to guide treatment approaches and initiate a more timely escalation of care for those ulcers not expected to heal in 12 weeks.

## Supplementary material

10.2196/69793 Checklist 1 STROBE checklist.

## Abbreviations

AI: artificial intelligence
DFUs: diabetes-related foot ulcers
RGB: red, blue, and green
STROBE: Strengthening the Reporting of Observational Studies in Epidemiology
WIfI: Wound, Ischemia, foot Infection
